# Implementation and Assessment of a Hybrid Training Course on Point-of-Care Pediatric Ultrasound in Vietnam During the COVID-19 Pandemic

**DOI:** 10.7759/cureus.45758

**Published:** 2023-09-22

**Authors:** Takaaki Mori, Yek Kee Chor, Thanh Liem Bui, Hong Anh Do, Gene Yong-Kwang Ong

**Affiliations:** 1 Department of Emergency Medicine, KK Women's and Children's Hospital, Singapore, SGP; 2 Department of Paediatrics, Sarawak General Hospital, Sarawak, MYS; 3 Department of Pediatrics, University of Medicine and Pharmacy, Ho Chi Minh City, VNM; 4 Department of Emergency, Critical Care, and Clinical Toxicology, University of Medicine and Pharmacy, Ho Chi Minh City, VNM; 5 Department of Children’s Emergency, KK Women’s and Children’s Hospital, Singapore, SGP

**Keywords:** emergency care, covid-19 pandemic, child, education, ultrasonography

## Abstract

Background

As point-of-care ultrasound (POCUS) has gained popularity, some educational guidelines have been developed. However, in Vietnam, no training course in pediatric POCUS has yet been developed. This was challenging, especially during the COVID-19 pandemic.

Objectives

This study aimed to implement a three-month hybrid training course for pediatric POCUS training in Vietnam using both online and face-to-face hands-on sessions and to assess participants’ self-efficacy level and change in their attitudes towards pediatric POCUS.

Methods

A hybrid training course in pediatric POCUS was implemented at a children’s hospital in Vietnam. This study developed a standardized training course, including online learning, live lectures, hands-on sessions, and skill assessment based on the POCUS consensus educational guidelines. Physicians interested in pediatric POCUS were recruited for participation. They completed a self-evaluation survey before and after the course using a Likert score to assess their background, self-efficacy in performing POCUS, overall satisfaction with the course, and change in their attitudes towards POCUS three months after the course.

Results

A total of 19 physicians participated in the course. The mean post-training self-efficacy score was significantly higher than the pre-course assessment score: 73.1 (standard deviation (SD): 7.2) vs. 48.9 (SD: 12.5) (p <0.05). The efficacy level was retained three months after the course. Furthermore, overall satisfaction with the course was high at 9.5 (SD: 0.6). After the course, almost all participants strongly agreed to increase the use of POCUS in their clinical practice.

Conclusion

A hybrid training course in pediatric POCUS was successfully implemented in Vietnam and found the participants’ self-efficacy level to be significantly higher after the course and the effect to be retained after the course. The training course could positively affect the participants’ attitudes towards POCUS, encouraging them to use POCUS more frequently in their clinical practice.

## Introduction

Point-of-care ultrasound (POCUS), a form of diagnostic and therapeutic ultrasound (US) performed by treating physicians, has gained popularity in pediatric emergency medicine (PEM) and pediatric intensive care [1]. This is because it provides real-time imaging that helps physicians’ clinical decisions without radiation exposure, which may cause secondary malignancies [2]. A consensus statement on pediatric POCUS was released by the American College of Emergency Physicians and the American Academy of Pediatrics in 2015 [3]. Additionally, in 2016, reporting guidelines for POCUS examinations were published [4]. Given its popularity, training courses in pediatric POCUS are being implemented worldwide, further confirming this technique’s efficacy [5-13].

Furthermore, due to the COVID-19 pandemic, the need for social distancing, and the restriction of face-to-face training, the POCUS courses have been cancelled despite the increasing demand. In this situation, most lectures and conferences have moved online or have combined face-to-face teaching with online tutorials [14]. Some studies have demonstrated the effectiveness of this teaching method [15-17].

Nonetheless, in developing countries in Southeast Asia, the utility of pediatric POCUS is limited, and learning opportunities for pediatric POCUS are insufficient due to the lack of educational resources and local teaching faculties; moreover, the COVID-19 pandemic has disrupted the plan to invite overseas faculties to teach on this. In addition, in Vietnam, neither structured educational courses nor any studies on pediatric POCUS have been documented. The aim of this study was to implement a hybrid training program for pediatric POCUS by adapting previously published consensus guidelines [3,4,18] and evaluating its effectiveness.

## Materials and methods

The present study was a quasi-experimental study investigating the self-efficacy level of participants in a hybrid pediatric POCUS training program conducted over three months between June and August 2022 in a children’s hospital in Vietnam and the change in attitudes towards pediatric POCUS after the training.

Pediatric POCUS training course

Instructors were recruited from Vietnam, Malaysia, and Singapore based on their previous experience in teaching POCUS at their respective institutions, each with at least three years of experience in using pediatric POCUS and having previously completed the pediatric emergency ultrasound course certified by the World Interactive Network Focused on Critical Ultrasound (WINFOCUS) [19]. They then established a curriculum for pediatric POCUS training through discussions following previously published educational guidelines [3,4,18], as shown in Table 1. The course was implemented from 1 June, 2022 to 7 August, 2022. The training consisted of recorded online lectures, a review of the logbook for assessing image acquisition, interpretation, and clinical application of POCUS, multiple choice questions (MCQs) of US applications for knowledge acquisition, case presentations of POCUS using a video conference system, and skill assessment in person (Table 2). The lectures dealt with cardiac US (focused cardiac ultrasound: FoCUS), lung US, US-guided procedures, EFAST (extended focused assessment of ultrasonography for trauma), and shock management (RUSH: rapid ultrasound for shock and hypotension, and HI-MAP scans). The MCQs consisted of questions covering knobology, cardiac US, lung US, US-guided procedure, EFAST, and shock management. Participants performed skill assessments by scanning patients admitted to a pediatric intensive care unit (PICU). Skills in knobology, cardiac US, lung US, and US for shock were assessed and marked as passing or failing by instructors using a previously prepared score assessment sheet (Table 3). 

**Table 1:** Learning objectives for pediatric POCUS

Skill Station	Minimum syllabus
Knobology	Explain and demonstrate the different transducer/probes available and each of their functions: curvilinear, linear, phased array and micro-convex. Explain the function of the button and function key on the ultrasound which include: power, patient data entry, pre-set, TGC (time gain compensation), B mode, M-mode, color flow, pulsed wave doppler, gain, depth, freeze, set, pause, measurement, scroll, cursor, print and media transfer button, reverse (switch screen indicator to be left and right of the screen and also inverse), and focus. To demonstrate each button and function usage on the model.
Basic lung ultrasound	Explain the setting and probe used for the examination. To demonstrate on the model the different zones of ultrasound examination. To demonstrate on the model longitudinal and oblique lung scan and identify the common structure in the standard lung field scan. To demonstrate the seven normal lung signs in an adult and child model, namely: bat wing sign, A line, B line, lung sliding, lung pulse, curtain sign, and sea-shore sign.
Basic ECHO view: Apical, parasternal, subcostal, and suprasternal	Explain the setting and probe used for the examination. To demonstrate on the model the common view, and structure identification of each view namely the apical 2 and 4 chambers, parasternal long and short axes, subcostal view, and suprasternal view. Identify the IVC, aorta, and hepatic vein.
Vascular	Explain the setting and probe used for the examination. To demonstrate on adult and child models the internal jugular, external jugular, subclavian vein, cephalic vein, basilic vein and femoral vein. To discuss the Seldinger technique and demonstrate ultra-sound-guided central line insertion on a chicken phantom.
EFAST and shock	Explain the setting and probe used for the examination. To demonstrate on the adult and child models the organs in the abdomen, namely the liver, gall bladder, portal vein, hepatic vein, IVC, aorta and urinary bladder. To demonstrate on the adult and pediatric models the sequences of EFAST. Explain the setting and probe used for the examination. To demonstrate to adults and children the sequence of the RUSH/HIMAP.

**Table 2:** Pediatric POCUS training program

Time	Content	Note
1^st^ June to 30 June 2022	Candidate prepares a logbook, including five case studies, according to the following five topics: Basic lung basic ECHO ultrasound-guided procedure EFAST ultrasound in shock	The logbook shall contain video images of the ultrasound with the history and demonstrate how POCUS assists in the clinical decision
30^th^ June 2022	Candidate to submit their logbook with five cases studied Candidate to choose one of the case to be present in the case study presentation on 16^th^ to 17^th^ July 2022	Candidate will send the logbook to the instructors
1^st^ July to 7 July 2022	Trainer will review logbook	Instructors will review the logbook for presentations. The instructors inform to all candidates regarding their results and feedback.
9 July 2022 (8AM Vietnam time)	MCQ and video clip question	An instructor organizes the exam via Google Form and Zoom
10 July to 15 July 2022	Candidate will prepare a case study presentation. The candidate must demonstrate how POCUS is used in clinical practice. The presentation will last less than 15 minutes. No mark will be given for a presentation lasting more than 15 minutes	
16^th^ July 2022 (Starting from 8 AM Vietnam time)	Candidate presents case study to the trainer. The candidate will be divided into two groups, and assessment will be done by the two groups of assessors	The presentation is performed using the Zoom platform (15 minute/case)
4^th^ to 5^th^August 2022	Skill station at VNCH: Stations are as bellowed: Knobology Heart Lung Shock the candidate will need to go through all the stations for assessment. Each cycle will accommodate four candidates, each candidate and will be given 15 minutes to demonstrate the skill in front of the examiner	
6^th^ to 7^th^August 2022	Advanced course at VNCH	Candidates who pass all exams are encouraged to join the advanced course

**Table 3:** Skill assessment form for pediatric POCUS

Basic cardiac			
Description	Total mark	Mark for candidate	Comment
Explain the setting and probe used for the examination	20		
To demonstrate on model the common view and structure identification of each view namely Apical 4 and 5 chamber, parasternal long short axis, subcostal view suprasternal view	60		
Identify IVC, aorta and hepatic vein	20		
Total mark	100		
EFAST and HIMAP			
Description	Total mark	Mark for candidate	Comment
Explain the setting and probe used for the examination	10		
To demonstrate on the adult and child model the organ in the abdomen namely liver, gall bladder, portal vein, hepatic vein, IVC, Aorta, urinary bladder	30		
To demonstrate on the adult and pediatric model the sequences of EFAST	30		
To demonstrate on the adult and child the sequence of the RUSH/HIMAP	30		
Total mark	100		
Basic lung			
Description	Total mark	Mark for candidate	Comment
To demonstrate on the model, the different zone of ultrasound examination	10		
To demonstrate on the model, the different zone of ultrasound examination	10		
To demonstrate on the model longitudinal and oblique lung scan and identify the common structure in the standard lung field scan	10		
To demonstrate the seven normal lung signs in adult and child model namely: Bat wing sign, A line, B line, Lung sliding, Lung pulse, curtain sign and sea- shore sign	70		
Total mark	100		
Vascular			
Description	Total mark	Mark for candidate	Comment
Explain the setting and probe used for the examination	20		
To demonstrate on adult and child model the internal jugular, external jugular, Subclavian vein, Cephalic vein, Basilic Vein and femoral vein	40		
To discuss the Seldinger technique and demonstrate ultra-sound guided central line insertion on chicken phantom	40		
Total mark	100		
Knobology			
Description	Total mark	Mark for candidate	Comment
Explain and demonstrate different transducer/probes available and each of their function: curvilinear, linear, phased array	20		
Explain and demonstrate the function of the button and function key on the ultrasound which include: Power, patient data entry, pre-set, TGC (time gain compensation), B-mode, M-mode, color flow, pulsed wave doppler, gain, depth, freeze, Set, measurement, scroll, print media transfer button reverse (switch screen indicator to be left and right of the screen and also inverse)	80		
Total mark	100		

Thereafter, another two days of advanced lectures and hands-on sessions were implemented, as shown in Tables 4, 5. The training consisted of tutorials, including 11 lectures (20 minutes each) and 10 hands-on training sessions (33-40 minutes each) with a trainer-to-learner ratio of 1:4-5. The lectures consisted of advanced cardiac US, including assessing cardiac function, airway/lung, esophagus/stomach, orbit, cranium, abdomen, musculoskeletal, and US-guided procedures. 

**Table 4:** Advanced pediatric POCUS training course (day 1)

Time	Topic
0800 to 0815	Registration
0815 to 0830	Welcome speech
0830 to 0900	Introduction
0900 to 0920	POCUS of orbit
0920 to 0940	POCUS Cranium
0940 to 1000	POCUS airway, esophagus and stomach
1000 to 1300	Hands-on orbit ultrasound group sequence (A – B – C) gene and Anh airway, esophagus, and stomach group sequence (B – C – A) Takaaki and Liem cranium group sequence (C – A – B) Chor
1300 to 1400	Lunch
1400 to 1420	POCUS in pathological lung and lung recruitment
1420 to 1440	Basic abdominal ultrasound
1440 to 1500	POCUS in pathological abdomen
1500 to 1520	Pericardiocentesis
1520 to 1700	Hands on POCUS pathological lung group sequence (A – B – C) Takaaki and Anh basic POCUS abdomen group sequence (B – C – A) Gene and Liem Pericardicentesis and procedure group sequence (C – A – B) Chor
1700 to 1715	Closing of day 1

**Table 5:** Advanced pediatric POCUS training course (day 2)

Time	Topic
0800 to 0820	Registration
0820 to 0900	Welcome: Q and A
0900 to 0920	POCUS in common childhood fracture
0920 to 0940	IVC pearl and pitfall: update in fluid responsiveness
0940 to 1000	POCUS in cardiac function
1000 to 1300	Hands-on POCUS in fracture group sequence (A – B – C ) Gene VTI, stroke volume and carotid artery pulse wave. Group sequence (B – C – A) Takaaki and Liem assessment of right heart pressure, diastolic function of left heart and IVC group sequence (C – A – B) Chor and Anh
1300 to 1400	Lunch
1400 to 1430	Use of POCUS in cardiac arrest
1430 to 1615	Back to patient: Head to toes Candidate go to bed side to practise ultrasound skill base on patient condition at PICU station 1: Gene sequence (1-2-3-4-5) station 2: Takaaki sequence (2-3-4-5-1) Station 3: Anh sequence (3-4-5-1-2) Station 4: Liem sequence (4-5-1-2-3) Station 5: Chor sequence (5-1-2-3-4)
1615 to 1645	Quiz and interactive session
1645 to 1700	Closing and end of course

For cardiac, airway/lung, and abdominal, orbital, and cranial US, children admitted to the PICU were used for scanning, and for US-guided procedures, a chicken leg was used as a hands-on model. The conference organizer explained the contents of the training course to the admitted patients who volunteered and obtained their oral consent before participation. 

Pre-prepared learning materials that demonstrated scanning techniques and normal and abnormal US images for each US application were provided to trainees before the start of the course. During the lectures, the instructors explained the learning objectives for each part and demonstrated normal and abnormal images. In the hands-on sessions, the instructors helped trainees scan live patients and/or simulators to practice their scanning skills. The instructors used standardized materials during the hands-on training to teach scanning techniques in each session, following each learning objective. They used video clips (cardiac, airway/lung, abdominal, ocular, and musculoskeletal US and US-guided procedures) or chicken legs (US-guided procedure) to illustrate abnormal images in each session. The course director monitored all stations to ensure a standard quality of instruction. 

Trainees

A course organizer recruited physicians interested in using pediatric POCUS as trainees in the hybrid pediatric POCUS training course. 

Questionnaire

The questionnaire used in our study was created by referencing previously published studies on implementing a POCUS training course [5,20,21] after discussion among instructors (Appendix). The overall satisfaction level with our training course and the trainees’ self-efficacy level in each US application before and after the course, the level of skill acquisition, and the change in behaviors towards pediatric POCUS after the training were selected for assessment. 

Data collection

Data were collected using printed materials by a primary investigator (TM), and trainees completed pre- and post-training evaluation forms. All assessment forms, including items on the participants’ background, such as their training level, experience participating in pediatric POCUS courses, experience using pediatric POCUS in daily practice, self-efficacy in pediatric POCUS use before and after the course, overall satisfaction with the course and the change in attitudes towards pediatric POCUS after the training, were provided in English (Appendix). The instructors were blinded by the written questionnaire. 

Measures

Self-efficacy and satisfaction rates were assessed using a Likert scale from 0 (no confidence) to 100 (complete confidence) with 11-point intervals measuring confidence [22] and a Likert scale from 0 (no satisfaction) to 10 (full satisfaction), respectively, after the training. Clinical skills in pediatric POCUS were assessed using a pre-prepared examination sheet for evaluating participants’ image acquisition, interpretation, and clinical application of POCUS in clinical practice. Changes in behavior regarding POCUS use and patient management in daily practice were assessed using a 5-point Likert scale (Appendix). 

Statistical analysis

SPSS Inc. Released 2009. PASW Statistics for Windows, Version 18.0. Chicago: SPSS Inc. was used for statistical analysis. Summary statistics are expressed as proportions with 95% confidence intervals (CI), and tests for association were performed using the paired t-test. An analysis of variance (ANOVA) was used to compare differences in the self-efficacy levels between pre- and post-training and three months after the training. P ≤ 0.05 was considered statistically significant.

## Results

**Figure 1:**
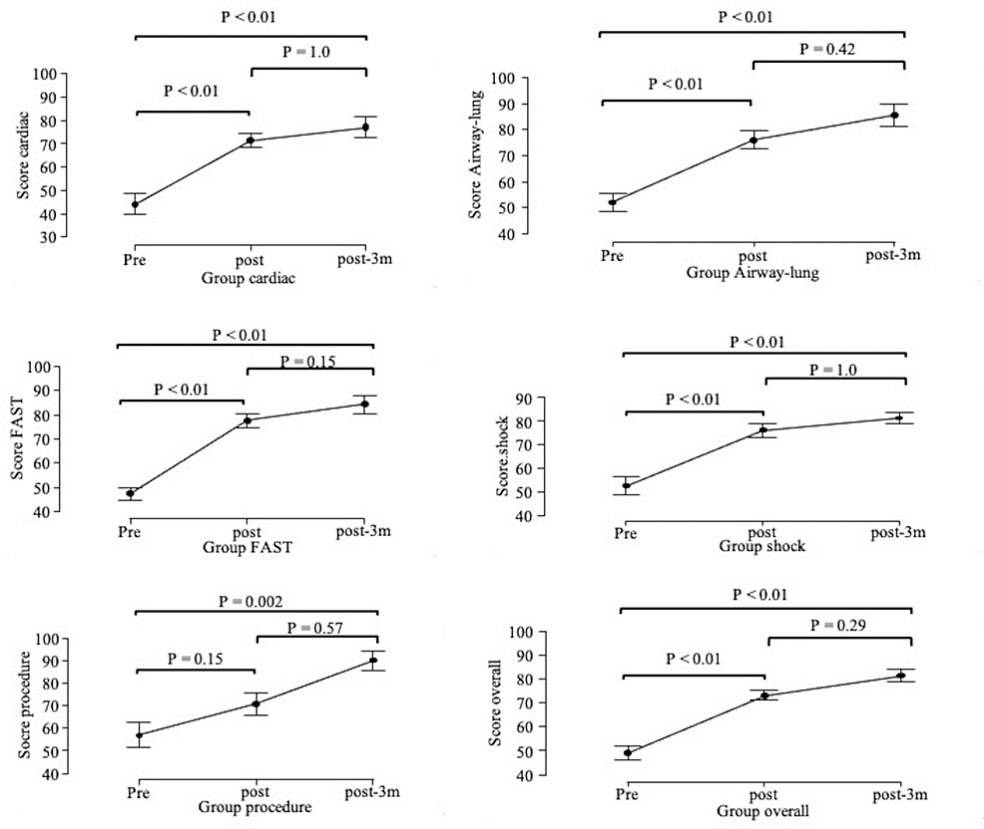
The self-efficacy level of pediatric POCUS in pre- and post-training courses

Of the 19 physicians enrolled, seven completed all the surveys. Table 6 shows their demographic characteristics. Seven participants were in post-graduate year (PGY) 1-5, five were in PGY 5-10, and seven were in PGY 11 or higher. Pediatricians comprised 78.9% of the trainees (15/19), followed by intensivists at 15.8% (3/19) and neonatologists at 5.3% (1/19). Fifteen participants (78.2%) had attended a POCUS training course, and 89.5% of the trainees responded that they routinely used ultrasonography. For skill assessment, all participants passed the examination for image acquisition and interpretation in all sections. The overall self-efficacy level in pediatric POCUS use after training was significantly higher than in the pre-course assessment: 73.1 (standard deviation (SD): 7.2) vs. 48.9 (SD: 12.5), respectively (p< 0.01) (Table 5). For each component, including cardiac, airway/lung, EFAST, and shock management, self-efficacy levels in the post-training evaluation were significantly higher. The self-efficacy level three months after the training remained unchanged from just after the training (Table 7, Figure 1). The overall satisfaction rate in the post-training evaluation was high at 9.5 (SD: 0.6). Participants’ attitudes towards pediatric POCUS were significantly positively affected. POCUS was used more frequently and became more useful in daily practice (mean 4.7 (SD: 0.45) and 4.7: 0.45) (Table 8).

**Table 6:** Characteristics of participants CI: confidence interval, PGY: post-graduate year, POCUS: point-of-care ultrasound

n=19	Percentage: 95% CI (%)
PGY (years)	
1-5	36.8 (19.0-59.1)
6-10	26.4 (115-49.1)
>10	36.8 (19.0-59.1)
Previous experiences in pediatric POCUS	78.9 (56.1-92.0)
Routine use in pediatric POCUS	89.5 (67.4-98.3)

**Table 7:** Survey results for pediatric POCUS (self-efficacy level of the participants) SD: standard deviation; FAST: focused assessment with ultrasonography for trauma

Self-efficacy level (n=19)					
Category	Pre-course (n=19) (Mean: SD)	Post-course (n=13) (Mean: SD)	3m-post-course (Mean: SD)	F-value (2.36)	p-value
Cardiac	44.2 (19.0)	71.5 (11.0)	71.5 (11.4)	16.7	< 0.01
FAST	47.4 (10.7)	77.7 (9.73)	77.7 (10.1)	47.9	< 0.01
Airway/lung	52.1 (14.7)	76.1 (11.5)	76.2 (11.9)	21.1	< 0.01
Shock	52.8 (15.9)	76.2 (10.8)	76.2 (11.2)	16.9	0.0001
Procedure	56.8 (23.6)	70.8 (16.4)	70.8 (10.1)	7.1	0.003
Overall	48.9 (12.5)	73.1 (7.2)	73.1 (7.5)	34.0	<0.0001
An increase in the usefulness of POCUS			4.7: 0.45		

**Table 8:** : Survey results for pediatric POCUS (satisfaction level and attitude toward POCUS in the post-training period) SD: standard deviation, POCUS: point-of-care ultrasound

Satisfaction level (n=13)	Mean: SD
Post-course satisfaction	9.5: 0.6
Attitudes for POCUS in post-training period (n=7)	Mean: SD
An increase in the frequency of POCUS use	4.7: 0.45
An increase in the usefulness of POCUS	4.7: 0.45

## Discussion

The present hybrid training course in pediatric POCUS was successfully implemented and demonstrated the high self-efficacy level of participants after the training. The training course was created based on previously published guidelines and consensus statements, including US applications and procedures that PEM and pediatric intensive care physicians are required to master [3,4,18]. A one-to-three-day training course on cardiac and lung US for pediatric intensivists and cardiologists substantially influenced participants’ knowledge and skill acquisition in performing US [7-10,12]. Other studies in Puerto Rico and Japan showed that a pediatric POCUS course in cardiac, pulmonary, and soft tissue US improved pediatricians’ knowledge and confidence level in pediatric POCUS. However, the sample size of this study was small [13,23]. Similarly, the present study demonstrated that comprehensive training in pediatric POCUS substantially improved participants’ self-efficacy. In settings like Vietnam, where no structured educational course on pediatric POCUS exists, the training course presented in our study has the potential to facilitate the use of pediatric POCUS by pediatric emergency and critical care physicians in clinical practice. 

The present hybrid POCUS training course combined online learning with in-person hands-on training and showed a high satisfaction rate and increased participants’ self-efficacy. The method may provide participants with learning opportunities on pediatric POCUS in Vietnam, where there is a shortage of teaching faculties on POCUS, and during the COVID-19 pandemic, when it is difficult to invite overseas lecturers. Online learning allows learners to watch recorded lectures and review them whenever they want, which is convenient and effective during the inconvenience caused by the COVID-19 pandemic. A study showed an online training course on US-guided nerve block in children increased physicians’ confidence in this procedure [6]. Another study demonstrated the non-inferiority of web-based learning to face-to-face education in knowledge acquisition of EFAST [15]. In addition, other studies demonstrated that online learning was non-inferior in terms of skill acquisition in the cardiac and musculoskeletal US [16,17]. A comparative study demonstrated no significant differences in knowledge acquisition between hybrid and traditional in-person POCUS training courses [24]. Therefore, online education is as effective as traditional in-person learning in pediatric POCUS training. 

Furthermore, the hands-on session in our study played an essential role in POCUS training. A study of pediatric POCUS courses showed that hands-on training increased self-efficacy towards POCUS among participants [23]. Another study reported the benefit of hands-on sessions for pediatric POCUS courses [24]. In our study, hands-on sessions with patients admitted to the PICU could provide a rich environment where participants could obtain pathologic images instead of using a US phantom or healthy volunteers. Many participants expressed satisfaction with the hands-on sessions. The participants’ self-efficacy level for pediatric POCUS use was high three months after the training. In addition, the use of POCUS significantly increased between pre- and post-course. Therefore, the results of the current study demonstrate our training course’s effectiveness in facilitating the use of pediatric POCUS.

From a medical and educational perspective, the Kirkpatrick model has been used to evaluate educational efficacy [25]. This model consists of levels 1 to 4, including trainees’ responses, knowledge, skill acquisition, changes in trainees’ behavior, and improvements in the quality of patient care [26]. The purpose of the questions in Supplement 1 is to ask, “Overall, are you satisfied with this course?” and “Are you confident of your skills in cardiac ultrasound?” were to assess the participants’ satisfaction level (level 1 criteria) and the self-efficacy level for each type of pediatric POCUS application (level 2 criteria), respectively. The significant improvement in self-efficacy scores in almost all categories demonstrated that the training satisfied the level 2 criteria. The purpose of a question in Supplement 1, “3 months after attending this course, have you used POCUS more frequently?” was to assess the application of the POCUS techniques taught in the course to participants’ daily practice (level 3). The result has the possibility of showing a positive change in applicability, although it is not enough to assess this component completely. Further assessment of levels 3 and 4 is required to assess the efficacy of the course in the future. 

The present study has several limitations. First, the trainees’ skill retention in pediatric POCUS was not assessed due to COVID-19 constraints. Second, the sample size of this study was small. This may have reduced the study’s statistical power. However, the impact was minimal, as demonstrated by the post-hoc analysis, which showed adequate power for the test. Third, participants in professional training courses tend to show low self-efficacy in the pre-test setting; thus, selection bias may have been included in the present study. Fourth, the Hawthorne effect may have influenced improvements in self-efficacy levels. The supervision of participants by the instructors and course director may result in an overestimation of their self-assessment in the post-training survey. Fifth, standardization, validity, and reliability of the questionnaire were not assessed, but the questions used in our study were followed by previously published studies showing the efficacy of POCUS training [5,20,21]. These assessments should warrant for further research. Sixth, the large number of participants previously had experience in POCUS, which might affect the relatively high self-efficacy level in the pre-training period and the slight difference between the pre-and post-training periods. 

## Conclusions

The present study showed the successful implementation of a hybrid training course in pediatric POCUS. The trainees’ self-efficacy level after the course significantly increased and remained high three months after the training. Furthermore, this study found that hybrid training could encourage physicians to use pediatric POCUS in clinical practice.

## Figures and Tables

**Figure 2:**
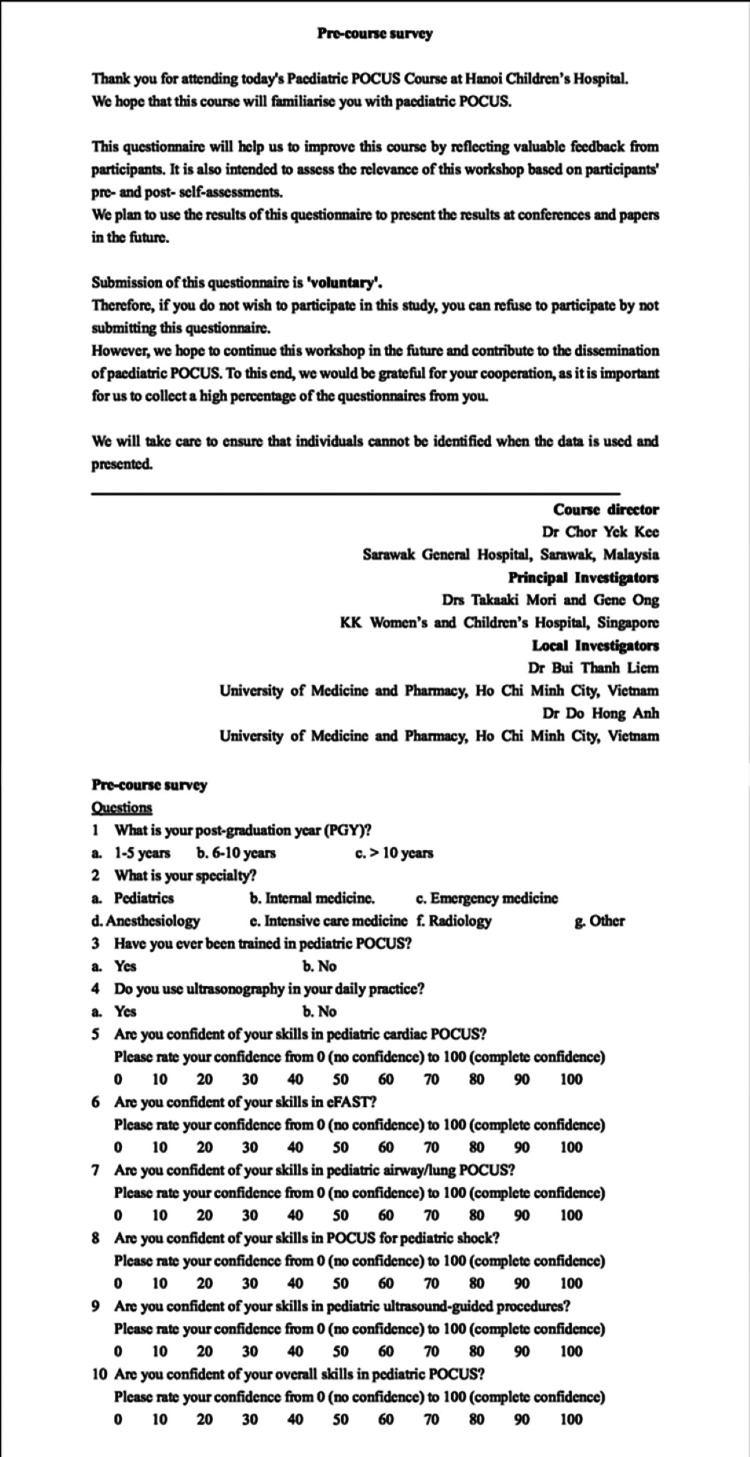
Pre-training survey

**Figure 3:**
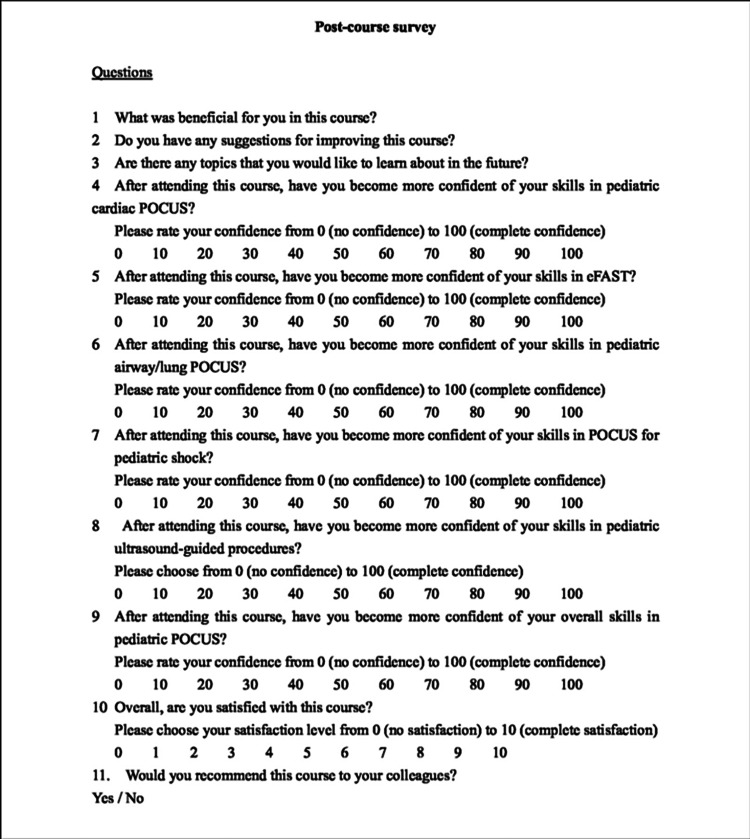
Post-training survey

**Figure 4:**
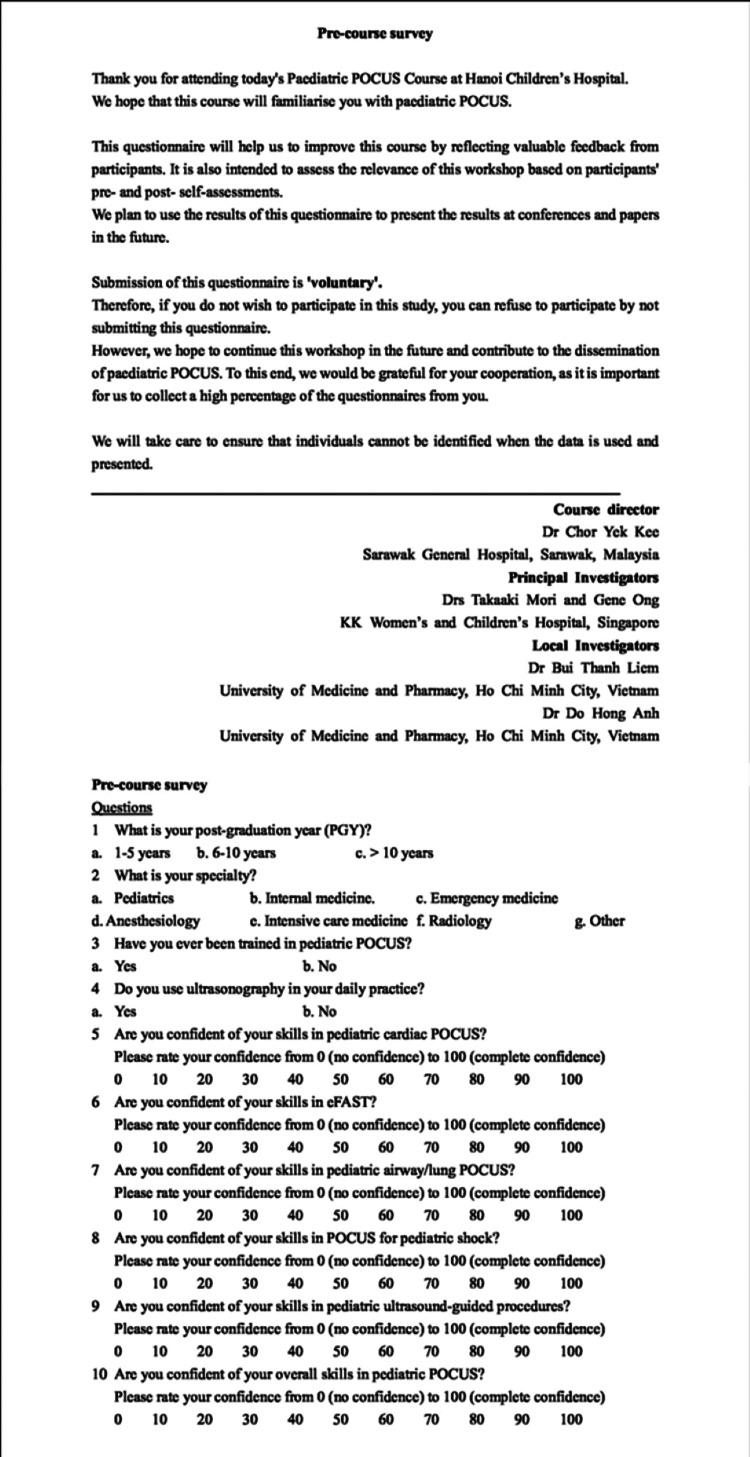
Pre-training survey

## Appendices

Questionnaire 

Figures 2-4 show the survey used.

## References

[1] Marin JR, Lewiss RE (2015). Point-of-care ultrasonography by pediatric emergency medicine physicians. Pediatr Emerg Care.

[2] Pearce MS, Salotti JA, Little MP (2012). Radiation exposure from CT scans in childhood and subsequent risk of leukaemia and brain tumours: a retrospective cohort study. Lancet.

[3] Abo A, Doniger S, Fischer J, Kessler D (2015). Point-of-care ultrasonography by pediatric emergency medicine physicians. Ped Eme Med Phys Pedi.

[4] Marin JR, Abo AM, Arroyo AC (2016). Pediatric emergency medicine point-of-care ultrasound: summary of the evidence. Crit Ultrasound J.

[5] Alzayedi AS, Azizalrahman AA, AlMadi HA, Althekair AM, Blaivas M, Karakitsos D (2017). Use and education of point-of-care ultrasound in pediatric emergency medicine in Saudi Arabia. J Ultrasound Med.

[6] Bretholz A, Doan Q, Cheng A, Lauder G (2012). A presurvey and postsurvey of a web- and simulation-based course of ultrasound-guided nerve blocks for pediatric emergency medicine. Pediatr Emerg Care.

[7] Ceresnak SR, Axelrod DM, Sacks LD, Motonaga KS, Johnson ER, Krawczeski CD (2017). Advances in pediatric cardiology boot camp: boot camp training promotes fellowship readiness and enables retention of knowledge. Pediatr Cardiol.

[8] Conlon TW, Himebauch AS, Fitzgerald JC (2015). Implementation of a pediatric critical care focused bedside ultrasound training program in a large academic PICU. Pediatr Crit Care Med.

[9] Gaspar HA, Morhy SS, Lianza AC (2014). Focused cardiac ultrasound: a training course for pediatric intensivists and emergency physicians. BMC Med Educ.

[10] Good R, Orsborn J, Stidham T (2018). Point-of-care ultrasound education for pediatric residents in the pediatric intensive care unit. MedEdPORTAL.

[11] Marin JR, Alpern ER, Panebianco NL, Dean AJ (2011). Assessment of a training curriculum for emergency ultrasound for pediatric soft tissue infections. Acad Emerg Med.

[12] Maskatia SA, Altman CA, Morris SA, Cabrera AG (2013). The echocardiography "boot camp": a novel approach in pediatric cardiovascular imaging education. J Am Soc Echocardiogr.

[13] Sepulveda-Ortiz V, Warkentine F, Starr-Seal R, Rominger A (2020). The effectiveness of a longitudinal ultrasound curriculum for general pediatricians working in a Puerto Rican emergency department: a pilot study. Ultrasound J.

[14] Nomura O, Irie J, Park Y, Nonogi H, Hanada H (2021). Evaluating effectiveness of YouTube videos for teaching medical students CPR: solution to optimizing clinician educator workload during the COVID-19 pandemic. Int J Environ Res Public Health.

[15] Platz E, Goldflam K, Mennicke M, Parisini E, Christ M, Hohenstein C (2010). Comparison of Web-versus classroom-based basic ultrasonographic and EFAST training in 2 European hospitals. Ann Emerg Med.

[16] Das D, Kapoor M, Brown C (2020). Comparison of hands-on versus online learning in teaching ultrasound skills for Achilles tendon rupture: a pilot study. Cureus.

[17] Canty D, Barth J, Yang Y, Peters N, Palmer A, Royse A, Royse C (2019). Comparison of learning outcomes for teaching focused cardiac ultrasound to physicians: A supervised human model course versus an eLearning guided self- directed simulator course. J Crit Care.

[18] Vieira RL, Hsu D, Nagler J, Chen L, Gallagher R, Levy JA (2013). Pediatric emergency medicine fellow training in ultrasound: consensus educational guidelines. Acad Emerg Med.

[19] (2023). WinFOCUS: Pediatric emergency & critical ultrasound course (PECUS). https://www.winfocus.org/course/pediatric-emergency-critical-ultrasound-course-pecus/..

[20] Nomura O, Wiseman J, Sunohara M, Akatsu H, Lajoie SP (2021). Japanese medical learners' achievement emotions: accounting for culture in translating Western medical educational theories and instruments into an Asian context. Adv Health Sci Educ Theory Pract.

[21] Yamada T, Minami T, Soni NJ, Hiraoka E, Takahashi H, Okubo T, Sato J (2018). Skills acquisition for novice learners after a point-of-care ultrasound course: does clinical rank matter?. BMC Med Educ.

[22] Bandura A (1977). Self-efficacy: toward a unifying theory of behavioral change. Psychol Rev.

[23] Mori T, Nomura O, Takei H, Fukuhara S, Ichihashi K (2022). Implementation and assessment of a pediatric point-of-care ultrasound training course in Japan: a pilot study. J Med Ultrason (2001).

[24] Janjigian M, Dembitzer A, Srisarajivakul-Klein C (2022). Design and comparison of a hybrid to a traditional in-person point-of-care ultrasound course. Ultrasound J.

[25] DeSilets LD (2018). An update on Kirkpatrick's model of evaluation: part two. J Contin Educ Nurs.

[26] Heydari MR, Taghva F, Amini M, Delavari S (2019). Using Kirkpatrick's model to measure the effect of a new teaching and learning methods workshop for health care staff. BMC Res Notes.

